# Present moment, past, and future: mental kaleidoscope

**DOI:** 10.3389/fpsyg.2014.00395

**Published:** 2014-05-01

**Authors:** Andrew A. Fingelkurts, Alexander A. Fingelkurts

**Affiliations:** BM-Science – Brain and Mind Technologies Research CentreEspoo, Finland

**Keywords:** subjective present moment, operation, subjective time flow, brain operational architectonics, EEG

It is the every person's daily phenomenal experience that conscious states represent their contents as occurring *now*. Following Droege (2009) we could state that consciousness has a peculiar affinity for *presence*. Some researchers even argue that conscious awareness necessarily demands that mental content is somehow held “frozen” within a discrete *progressive present moment* (James, 1890; Lynds, 2003). Thus, phenomenal content seems to be minimally conscious if it is integrated into a single and coherent model of reality during a *“virtual window” of presence* (Metzinger, 2003; see also Brown, 1998; Varela, 1999; Smythies, 2003).

In order to explain such features of consciousness as phenomenal unity and continuity within the *current present* along with a succession of discrete thoughts that give rise to feeling of the past and future, a reference to mechanisms outside the phenomenal realm is necessary (Revonsuo, 2003). Thus, the question of what could be the neurophysiological mechanisms responsible for these experiences should be addressed.

In this Opinion Article we shall build our argument based on the biological realism approach to consciousness proposed by Revonsuo (2006). According to this approach, subjective consciousness is a real phenomenon that is tightly anchored to a biological reality within the human brain. Broadly speaking, the human brain is the specific physical “location,” where the subjective mental reality and the objective neurobiological reality are intimately connected along a unified metastable continuum (Fingelkurts et al., 2009, 2013).

We have argued previously (Fingelkurts et al., 2010) that phenomenal consciousness refers to a higher level of organization in the brain and captures all *immediate* and undeniable (from the first-person perspective) phenomena of subjective experiences (hearing, seeing, touching, feeling, embodiment, moving, and thinking) that present to any person *right now* (subjective present) and *right here* (subjective space). By this definition even remembering the past images and planning the future events can't be performed other than in the *present moment* and in relation to current state of affairs (see also Lynds, 2003; Droege, 2009). This is so because someone possesses phenomenal consciousness if there is any type of subjective experiences that is *currently present* for him/her (Fingelkurts et al., 2010).

In this context what is presented as *now* is not simply whatever sensory or other representations occur in the brain at any given moment but rather the spatial-temporal hierarchy of selected and nested metastable states of neuronal assemblies that serve in real time as a basis for the subjective experiences of the “present moment.” Among many theories, the Operational Architectonics (OA) theory of brain and mind functioning (Fingelkurts and Fingelkurts, 2001, 2008; Fingelkurts et al., 2010, 2013) explicitly utilizes the hierarchy of nested metastable states of neuronal assemblies. In short, OA theory is centered on the notion of *operation*. Operation is broadly defined as the process or state of being in effect and it has a beginning and an end (Collins Essential English Dictionary, 2006). In fact, everything which can be represented by a process is an operation. The notion of operation plays a central role in bridging the brain-mind gap and makes it possible to identify what at the same time belongs to the mental level and to the neurophysiological level of brain activity organization, and acts as a mediator between the two (Fingelkurts and Fingelkurts, 2001, 2008; Benedetti et al., 2010). Understanding of the operation as a process and considering its combinatorial nature, seems especially well suited for describing and studying the mechanisms of how information about the objective physical entities of the external world can be integrated within the *present moment* in the internal subjective domain by means of entities of distributed neuronal assemblies (Fingelkurts et al., 2010, 2013). In line with this conceptualization, simple cognitive operations that present some partial aspect of the whole object/scene/concept are presented in the brain by local 3D-fields produced by discrete and transient neuronal assemblies, which can be recorded by an electroencephalogram (EEG) (Figures 1A,B). More complex operations that constitute the whole object or scene are brought into existence by joint (synchronized) simple operations in the form of coupled 3D-fields—so called operational modules (OMs) of varied complexity (Fingelkurts and Fingelkurts, in press). Further synchronization of several OMs (complex field spatial-temporal patterns; Figure 1A) forms even more coarse scales of nested functional hierarchy (Feinberg, 2000) that is now able to present and hold highly complex sensorial inputs as coherent perceptions of the world, create internal complex images and form conscious decisions (Fingelkurts et al., 2010, 2013). The recombination of neuronal assemblies and their operational modules into new configurations gives rise to a nearly inexhaustible source of presenting different qualities, patterns, objects, scenes, concepts and decisions.

**Figure 1:**
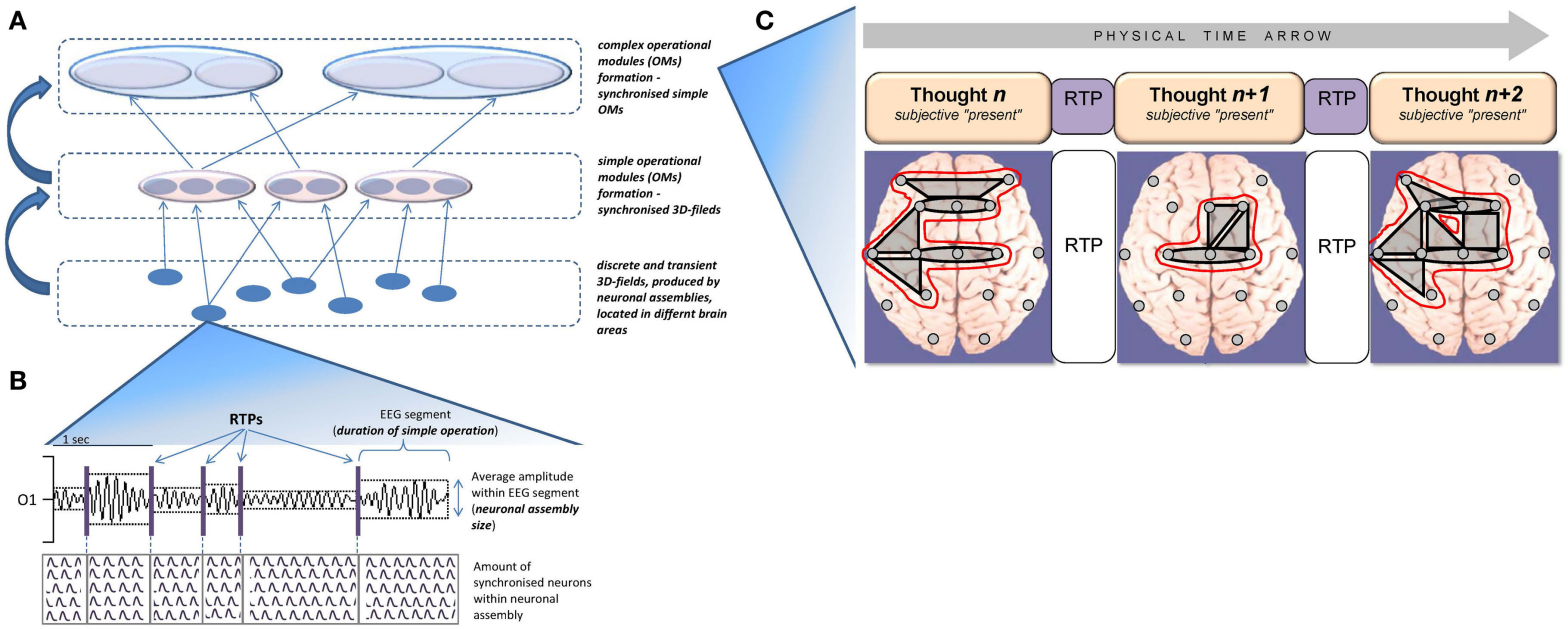
**Schematic representation of the nested functional hierarchy of spatiotemporal patterns of 3D electromagnetic fields produced by neuronal assemblies and operational modules formation, as well as their dynamics. (A)** In a nested hierarchy, higher levels are physically composed of lower levels, and there is no central control of the system resulting in weak constraint of higher upon lower levels. **(B)** Illustration of the neuronal assembly's dynamic and its relation to the EEG signal segments (for methodological details see Fingelkurts and Fingelkurts, 2008, in press); RTP, rapid transitional processes. Local EEG signal (O1-left occipital location) is filtered in alpha (7–13 Hz) frequency band. **(C)** Diagram depicting dynamics of operational modules (OMs). Phenomenological level of description illustrates the ever-changing stream of consciousness, where each momentarily stable pattern is a particular kaleidoscopic image separated from one another by the transitive fringes (or rapid transitional periods; RTPs). Neurophysiological level is presented by a relatively stable complex OMs (outlined by the red line), that undergo abrupt changes simultaneously with changes in phenomenological level. Such abrupt changes marked as rapid transitional periods (RTPs). Gray shapes illustrate simple OMs. This scheme is based on data published in Fingelkurts et al. (2003). Methodological aspects of how 3D electromagnetic fields and their combination in the form of operational modules are extracted from EEG could be found in Fingelkurts and Fingelkurts (2008, in press).

In the following we will discuss how the OA framework could implement the subjective present and some other temporal phenomena. We argue that at the phenomenological level, the lasting OM would be experienced as the “phenomenal present” of consciousness (Figure 1C). This hypothesis remains to be proven experimentally, however some empirical evidence already exists. For example, the mean duration of OMs (derived from an EEG with a frequency band of 0.3–30 Hz) usually varies from 80–100 ms for large OMs spanning the cortex to 30 s for small local OMs. These accounts, including duration variation, are consistent with known estimates for the *frame of a specious present*, which varies from ~100 ms to several seconds depending on circumstances (Pöppel, 1988).

However, if the brain could implement only a complex but static OM, then such a brain would only experience the presence of one unified world frozen into an internal *now* (Metzinger, 2003). Neither the complex texture of subjective time flow, nor true perspectivalness that goes along with a first-person point of view would exist in such situation (Fingelkurts et al., 2010). Therefore, a dynamic succession of phenomenal moments that are integrated into the flow of subjective time is needed. Indeed, as it is evident from the first-person perspective, the actualization of full-fledged phenomenal objects, images or scenes is realized on a “one-at-a-time” basis, moving serially from one phenomenal pattern within a specious present to another (Revonsuo, 2006). This process gives rise to a stream of consciousness that is best conceptualized in the James' metaphor of a *kaleidoscope* (James, 1890). Using this metaphor James illustrates the ever-changing stream of thoughts like a rotating kaleidoscope where each *momentarily* stable pattern constructed from multiple pieces (local fields in our interpretation) is a speciously presented thought (OM in our interpretation). Thus, the succession of phenomenal images or thoughts is neurophysiologically presented by the succession of discrete and relatively stable OMs, which are separated by rapid transitive processes (RTP), i.e., abrupt changes of OMs (Figure 1C). As it has been shown experimentally, at the critical point of transition in mental states, the OM undergoes a profound reconfiguration which is expressed through the following process (Fingelkurts et al., 2000, 2003; Fingelkurts and Fingelkurts, in press): The OM, which is comprised from a set of local bioelectrical fields produced by transient neuronal assemblies across several brain areas, rapidly loses functional couplings and establishes new couplings within another set of local bioelectrical fields, thus demarcating a new OM in the volumetric operational space-time continuum of the brain (Figure 1C).

Thus, the presented model for OM mediated succession of phenomenal images or thoughts is one way of understanding how *subjective time flow* is mentally (re)constructed beyond the phenomenal horizons of “presence.” Subjective time flow is not actually experienced or “perceived,” rather it emerges as the product of cognitive higher-order processes operating on the OMs (Fingelkurts et al., 2010). Such higher-order processes are also expressed in the form of complex OMs, that not only superceedes lower-level OMs, but also execute memory consolidation and retrieval operations (Fingelkurts et al., 2003). Given such a mechanism, the variation in *subjectively experienced speed of time* could be also explained. When the OMs' average duration decreases, there are many more OMs managing to sequence each other within a given time unit. We suggest that this overflow of OMs would be commonly experienced as an acceleration of the subjective time. Conversely, if the average duration of OMs was to increase, then the subjective experience of time would slow down. Below, we review some experimental evidence in support of our theorizing.

It is well known that certain psychoactive agents create subjective time distortions when administered. For example, opioids can be used to prolong the subjectively perceived duration of thought (Galski et al., 2000). In agreement with OA framework, it has been shown that opioids do indeed increase the duration of the life-span of neuronal assemblies (indexed by EEG quasi-stationary segments) and limit the synchronization between their operations, thus reducing the possible number of OMs while increasing their life-span (Fingelkurts et al., 2006).

Another important model, where subjective experience could be easily manipulated is hypnosis. In a neutral hypnotic state the subject experiences an altered background state of consciousness different from the normal baseline state of consciousness without the need of suggestion (Kallio and Revonsuo, 2003). This subjective state is characterized by “emptiness” or “absorption” brought about by dissociations in the cognitive modules that are temporarily incapable of normal communication with each other (Gruzelier, 2000). Additionally, it has been shown that the subjective sensation for the passage of time is stretched during hypnosis, because internal events are subjectively slowed (Von Kirchenheim and Persinger, 1991; Naish, 2001). Adhering to the tenets of OA framework, these subjective experiences should be reflected in the operational architectonics of the electromagnetic brain field. It was indeed shown that the functional life-span of neuronal assemblies (indexed by the EEG quasi-stationary segments) was significantly *longer* during hypnosis when compared with the normal/baseline conscious condition (Fingelkurts et al., 2007). It was further found that the number and strength of synchronized operations among different neuronal assemblies were significantly *lower* during hypnosis than during the baseline, thus limiting the possibility for any OMs to emerge. As a result they were absent (Fingelkurts et al., 2007). Since OMs represent the formation of integrated conscious experiences, their absence may explain such unusual subjective experiences during hypnosis as amnesia, timelessness, detachment from the self, a “willingness” to accept distortions of logic or reality, and the lack of initiative or willful movement (Dietrich, 2003).

Dreaming is a special case where the phenomenal world is realized in the brain in its “pure form,” because it is nearly completely isolated from the external physical world and the rest of the body. Dreams can appear in REM as well as in the nonREM sleep (Nir and Tononi, 2010). However, the nature of dreams in REM and nonREM sleep is different: during REM the dreams are complex, organized, *temporally evolving*, multimodal, and often bizarre (Hobson et al., 2000), while in nonREM the dreams are characterized by simple, *static* or isolated image(s) or though(s), usually of one modality (Noreika et al., 2009). The OA prediction is that nonREM dreams should be accompanied by short-lived small neuronal assemblies and long-lived large neuronal assemblies, and by the significant increase of operational synchrony (poor set of OMs) among different neuronal assemblies in order to subjectively present static images or thoughts. In a pilot nonREM sleep study (Fingelkurts and Fingelkurts, in press) we found that nonREM dreams were indeed accompanied by the small short-lived and large long-lived neuronal assemblies, as well as significant operational synchrony increase in the OA organization of the brain. Future research should establish the OA data for REM sleep dreams.

This brief review of results supports the suggested neurophysiological mechanism (within the operational architectonics of the human brain field) responsible for the experiences of the “present moment,” past, and future.

## Conflict of interest statement

The authors declare that the research was conducted in the absence of any commercial or financial relationships that could be construed as a potential conflict of interest.
